# Carbapenemase-Producing Klebsiella pneumoniae: A Comprehensive Review of Epidemiology, Resistance Mechanisms and Phenotypic Detection

**DOI:** 10.7759/cureus.110209

**Published:** 2026-06-03

**Authors:** Aishwarya S Kadam, Geeta Karande, Satish R Patil

**Affiliations:** 1 Department of Microbiology, Krishna Institute of Medical Sciences, Krishna Vishwa Vidyapeeth (Deemed to Be University), Karad, IND

**Keywords:** carbapenemase production, carbapenem resistance, klebsiella pneumoniae, modified carbapenem inactivation method, modified hodge test (mht), multidrug resistance (mdr)

## Abstract

Carbapenem-resistant *Klebsiella pneumoniae* is a major global health concern associated with severe infections, high mortality, and limited treatment options. This review provides a comprehensive overview of the epidemiology, pathogenesis, antimicrobial resistance mechanisms, treatment strategies, and diagnostic approaches for carbapenemase-producing *Klebsiella pneumoniae* isolates from clinical samples. Particular emphasis is placed on phenotypic detection methods, including the modified Hodge test, modified carbapenem inactivation method, carbapenemase Nordmann-Poirel test, and inhibitor-based assays, along with their advantages and limitations. The review also highlights emerging molecular diagnostics and their role in improving rapid detection and infection control. Overall, the review emphasizes the continued importance of phenotypic assays, particularly in resource-limited settings, while acknowledging the expanding role of advanced diagnostic technologies.

## Introduction and background

*Klebsiella pneumoniae* is a Gram-negative, rod-shaped, and non-motile bacterium. It belongs to the family Enterobacteriaceae. It is a facultative anaerobic bacillus. The members of the genus *Klebsiella are *commonly found in soil, water, and the human intestine; also, about 30% of strains can fix nitrogen in anaerobic conditions.* Klebsiella pneumoniae* is an opportunistic pathogen that colonizes the mucosal surface of the oropharynx and gastrointestinal tract. It stands out as a key opportunistic pathogen behind numerous healthcare-associated infections, including bloodstream infections, pneumonia, urinary tract infections, and wound infections [1].

Enterobacteriaceae antimicrobial resistance has significantly grown in recent years. Beta-lactams, aminoglycosides, fluoroquinolones, and carbapenems are frequently used antimicrobial agents, as these organisms have become resistant to them. Therefore, reduced therapeutic options, increased treatment expenses, and prolonged hospital stays and mortality are all caused by drug-resistance concerns [1]. Many virulence factors, including capsular lipopolysaccharides (LPS), fimbrial and non-fimbrial adhesins, siderophore, and the capability to produce biofilms, contribute to the pathogenicity of *Klebsiella pneumoniae* and other species. The infection becomes more difficult due to its capacity to create biofilms. Among the biggest obstacles to treating *Klebsiella* *pneumoniae* infection is the establishment of carbapenemase. The infections produced by Enterobacteriaceae that exhibit multidrug resistance are frequently treated with carbapenems. Over the past decade, carbapenem-resistant Enterobacteriaceae, particularly carbapenemase-producing *Klebsiella pneumoniae*, have emerged as a major global public health threat with rapidly increasing prevalence worldwide. Public health and clinical patient care are at risk from the quick development and spread of these enzymes. These enzymes generate resistance against nearly all β-lactamase agents, such as carbapenems, cephalosporins, penicillin, and monobactams. Therefore, this review aims to summarize and critically discuss the current knowledge on carbapenemase-producing *Klebsiella pneumoniae*, including its epidemiology, resistance mechanisms, diagnostic approaches, treatment strategies, and emerging developments reported in the literature* *[2].

## Review

Discovery

In 1882, Carl Friedlander isolated the bacterium in pure culture, and it was initially referred to as “Friedlander’s bacillus” [3]. The genus *Klebsiella *was later named in honor of “Edwin Klebs” (1834-1913), who had earlier identified the organism in the lung tissues of pneumonia patients in 1875 [4]. Subsequently, Hans Christian Gram developed the Gram staining technique in 1884 to improve the visualization of bacteria in tissue samples. This method was especially beneficial in differentiating *Streptococcus pneumoniae* (*pneumococcus*) from *Klebsiella pneumoniae *in cases of pneumonia [5,6].

Morphology

It is a short, plump, straight, capsulated Gram-negative, rod-shaped bacillus. The organism is non-motile and measures approximately 1-2 µm in length and 0.5-1.8 µm in width. In Gram-stained smears, the mucopolysaccharide capsule is visible as a distinct halo surrounding the rods [1]. 

Antigenic properties

The synthesis of capsular polysaccharides, LPS, and fimbriae and unique iron acquisition mechanisms are some of the elements that contribute to the antigenic pathogenicity of *Klebsiella*, especially *Klebsiella pneumoniae*. Furthermore,* Klebsiella pneumoniae *can create biofilms that strengthen its resistance by shielding it from antimicrobial agents and host immune systems [7]. Unlike certain bacteria, *Klebsiella* does not create exotoxins but does have endotoxins that contribute to its pathogenicity. The three primary antigens found in capsulated *Klebsiella* organisms are somatic smooth (O-antigen), somatic rough (R-antigen), and capsular (K-antigen) [8]. The R-antigen is protein-based, while the K- and O-antigens are carbohydrate-based. *Klebsiella *species are classified into approximately 80 serotypes based on capsular (K) antigens. Types 1-6 are most frequently identified in the human respiratory system. The capsular "swelling" reaction, counter-current immuno-electrophoresis, and enzyme-linked immunosorbent assay are common techniques used to detect capsular antigens [9].

Environmental resistance

*Klebsiella* are able to survive for many months at surrounding temperatures, demonstrating a relatively high capacity for environmental persistence. However, the bacteria are destroyed within approximately one hour when exposed to heat at 65 °C. They are sensitive to commonly used disinfectants such as phenol and chloramine solutions. The virulence of *Klebsiella* is closely linked to the presence of its capsule, which protects the organism from host immune defense. When the bacteria lose their capsule and enter the host, they become non-virulent and are rapidly phagocytosed. In contrast, highly pathogenic capsulated strains are capable of spreading to multiple organs and can cause death in experimental animals, such as mice, within 24 to 48 hours [10].

Carbapenem resistance

Carbapenem resistance in Klebsiella pneumoniae occurs through multiple mechanisms. The primary mechanism is carbapenemase production, including enzymes such as Klebsiella pneumoniae carbapenemase (KPC), New Delhi metallo-β-lactamase (NDM), OXA-48, Verona integron-encoded metallo-β-lactamase (VIM), and imipenemase metallo-β-lactamase (IMP), which hydrolyze carbapenem antibiotics and render them ineffective. Resistance may also result from the combined effect of extended-spectrum β-lactamase (ESBL) or AmpC β-lactamase production with porin loss, reducing antibiotic entry into the bacterial cell. Additionally, overexpression of efflux pumps contributes to resistance by actively removing antibiotics from the cell. These mechanisms collectively increase multidrug resistance and complicate treatment strategies [10,11].

Molecular Mechanisms of Carbapenem Resistance

Carbapenems, including imipenem, meropenem, and ertapenem, are broad-spectrum β-lactam antibiotics that are commonly used as last-line methods of treatment for severe infections due to multidrug-resistant (MDR) Gram-negative bacteria. However, the development of carbapenem resistance has become a significant global health issue. This resistance most frequently arises due to the formation of carbapenemase enzymes capable of breaking down carbapenem antibiotics and makes them ineffective. Among Gram-negative organisms, carbapenem-producing Enterobacterales, especially *Klebsiella pneumoniae*, have been classified as critical priority pathogens according to the World Health Organization because of their rapid dissemination and the limited therapeutic options available [12].

Classification

Several important carbapenemase enzyme classes are linked with carbapenem antibiotic resistance in *Klebsiella pneumoniae* as follows.

Class A Carbapenemases

These include KPC, among the most widely distributed enzymes, commonly reported across the United States, Europe, and China. These enzymes are generally plasmid-mediated, which enables easy transfer between bacterial strains, and they are capable of hydrolysing almost all β-lactam antibiotics.

Class B Carbapenemases

Class B carbapenemases are known as metallo-β-lactamases and include enzymes such as NDM, VIM, and IMP. Zinc ions are required by these enzymes for their activity and are not inhibited by certain β-lactamase inhibitors, making the infections they cause difficult to manage. Among these, NDM is particularly common in India and has spread across the world.

Class D Carbapenemases

These include Oxacillinase-48 (OXA-48)-like enzymes, which are highly prevalent in regions such as the Middle East, North Africa, the Mediterranean area, and India. These enzymes often produce relatively low levels of resistance, which may lead to difficulty in their detection more challenging in routine laboratory tests [13]. 

Other mechanisms

Other mechanisms apart from carbapenemase production also have a role in producing carbapenem resistance in *Klebsiella pneumoniae*. These include the generation of ESBLs or AmpC β-lactamases together with the loss or alteration of outer membrane porins such as OmpK35 and OmpK36, which decreases the entry of antibiotics into the bacterial cell. Increasing the production of efflux pumps can also export antibiotics from the cell, further lowering drug susceptibility. In addition, resistance genes are typically passed through horizontal gene transfer mediated by plasmids, integrons, and transposons, promoting the rapid spread of carbapenem resistance among bacterial populations in healthcare settings [14].

Phenotypic methods for carbapenemase detection

Phenotypic methods are still commonly used to detect carbapenemase production because they are cost-effective and easy to perform in routine clinical laboratories. These techniques generally rely on the ability of carbapenemases to break down carbapenem antibiotics or on their inhibition by specific agents. Carbapenemase production among the bacteria can be identified by performing various phenotypic methods, as listed in Table 1.

**Table 1:** Phenotypic detection methods for carbapenemase detection MHT: modified Hodge test [1]; mCIM: modified carbapenem inactivation method [13];  Carba NP test: Carbapenemase Nordmann-Poirel test [11]; CDT: combined disc test [1]

Phenotypic Test	Key Features & Importance	Efficacy & Performance	Limitations
Modified Hodge test (MHT)	Traditionally used as a primary test, this uses an indicator strain to form a cloverleaf pattern on carbapenem hydrolysis.	Highly sensitive for recognizing Class A carbapenemases such as KPC.	Frequently produces false positive results (often due to ESBL/AmpC with porin mutations) and has lower sensitivity for metallo-β-lactamase (MBLs) detection.
Modified carbapenem inactivation method (mCIM)	A CLSI-recommended standard method involving the incubation of a meropenem disk in a bacterial suspension.	Highly specific and sensitive for identifying a broad range of carbapenemase enzymes, e.g., KPC, NDM, VIM, IMP, and OXA-48.	The test requires overnight incubation, resulting in a slower turnaround time compared to other detection tests.
Carba NP test	This is a rapid biochemical test that detects the in vitro hydrolysis of imipenem through visually observable pH-driven colour changes.	Highly specific and fast, it often gives more accurate results in a short time period.	This may show slightly lower sensitivity when detecting certain OXA-48-like enzymes, which possess weak hydrolytic activity.
Combined disc test (CDT)/inhibitor-based assays	This test uses specific chemical inhibitors like phenylboronic acid (PBA) and EDTA to target and inhibit specific enzyme classes.	Helpful for both detecting carbapenemase production and classifying the specific enzyme type (e.g., distinguishing KPC from MBLs).	Requires multiple disks and specific inhibitors, which can increase the complexity of the setup and result interpretation.

Epidemiology

The rise of MDR pathogens, particularly carbapenem-resistant Klebsiella pneumoniae (CRKP), has become a major global public health concern due to the reduced effectiveness of last-line antibiotics such as carbapenems. According to recent WHO and global surveillance reports, carbapenem-resistant Enterobacteriaceae are increasingly prevalent in healthcare settings worldwide, with particularly high resistance rates reported in South Asia, including India, Pakistan, and Bangladesh. In India, NDM-producing Klebsiella pneumoniae remains one of the most commonly reported carbapenemase-producing strains in tertiary care hospitals and intensive care units (ICUs) [15].

Carbapenemase-producing Klebsiella pneumoniae is frequently associated with healthcare-associated infections, especially in ICUs, long-term care facilities, and immunocompromised patients, where both endemic transmission and hospital outbreaks have been widely documented. Major carbapenemase enzymes include KPC, NDM, and OXA-48, all of which contribute to resistance against carbapenem antibiotics. While healthcare-associated spread remains the predominant mode of transmission, increasing reports of community-associated infections have also emerged in recent years, raising concerns regarding broader dissemination outside hospital settings. The continued spread of these resistant strains has significantly increased morbidity, mortality, healthcare costs, and therapeutic challenges worldwide [16].

Pathogenesis and spread

*Klebsiella pneumoniae* is frequently isolated from clinical specimens and is known to cause a classical form of primary pneumonia. It is rarely present in the oropharynx of healthy individuals, with a carrier rate of about 1-6%; however, the prevalence may increase to nearly 20% among hospitalized patients. This type of colonization can lead to lung infections, particularly in individuals with underlying conditions such as alcoholism, diabetes mellitus, and chronic obstructive pulmonary disease. Pneumonia caused by *Klebsiella pneumoniae* is usually severe and destructive, often involving extensive necrosis and hemorrhage, leading to the production of sputum that may appear thick, mucoid, and brick-red or thin with a “currant jelly-like” appearance. In severe cases, complications such as lung abscesses, chronic cavitary lesions, internal hemorrhage, and hemoptysis may occur. Pleuritis is also frequently observed, which explains the detection of pleuritic chest pain in nearly 80% of patients. Apart from respiratory infections, *Klebsiella pneumoniae *can cause several extrapulmonary infections, including enteritis and meningitis in infants, urinary tract infections in both children and adults, and septicemia.

A new hypervirulent clinical variant of *Klebsiella pneumoniae* known as hypervirulent *Klebsiella pneumoniae* (hvKP) has emerged during the past two decades. The first reports were mainly from countries in the Asia-Pacific, such as Taiwan, South Korea, Vietnam, and Japan, where a distinct clinical syndrome of community-acquired *Klebsiella pneumoniae* infection was described. These infections commonly presented as pyogenic liver abscesses and showed a high tendency for metastatic spread to distant organs. In recent years, an increasing number of cases have likewise been documented in North America, South America, the Caribbean, and other parts of the world. This hypervirulent strain is recognized by several clinical and phenotypic features, including (a) its ability to lead to infections in otherwise healthy, ambulatory individuals; (b) occurrence of infections at unusual sites such as the liver, eye, and cerebrospinal fluid; (c) a strong capacity for metastatic dissemination; and (d) the formation of hypermucoviscous colonies when cultured on agar plates. 

The hypermucoviscous phenotype can be demonstrated using the String test. A positive result occurs when a bacteriological loop or needle touches the bacterial colony and is lifted above the agar surface, producing a viscous string greater than 5 mm in length. Because of the distinctive and severe clinical features associated with these strains, laboratories are encouraged to report *Klebsiella pneumoniae* isolates showing a positive string test as hypervirulent *Klebsiella pneumoniae* [17,18]. 

Clinical significance

The second most common aerobic bacterial flora in the human gut is *Klebsiella pneumoniae*, which has developed into a significant contributor to infections linked to healthcare. Infections like pneumonia, catheter-associated urinary tract infections, ventilator-associated pneumonia, surgical site infections, bloodstream infections, septicemia, and several pyogenic infections are frequently linked to it. In rare instances, it may also result in diarrhea, conjunctivitis, and opportunistic infections. *Klebsiella pneumoniae*, *subspecies pneumoniae,* is the most pathogenic of its subspecies. It can cause severe lobar pneumonia, urinary tract infections, neonatal meningitis, septicemia, and different pyogenic diseases like abscesses and wound infections [19,20].

Nosocomial infections are frequently caused by *Klebsiella pneumoniae,* which often colonizes the oropharynx of hospitalized individuals. Pneumonia is most frequently linked to serotypes one, two & three, and in roughly 25% of cases, positive blood cultures are produced. High death rates, especially in neonatal sepsis, are caused by the antibiotic resistance of many hospital pathogens. This bacterium frequently causes severe and devastating pneumonia, resulting in thick, mucoid, brick-red sputum that occasionally seems thin and has a consistency similar to currant jelly. Rarely, some strains have been identified to produce a heat-stable enterotoxin akin to that of *Escherichia coli*, and they may also induce diarrhea [21].

Two major kinds of *Klebsiella pneumoniae* have been identified: hypervirulent *Klebsiella pneumoniae* (hvKP) and classical *Klebsiella pneumoniae* (cKP). cKP isolates, which frequently include antibiotic-resistant clones that occur widely around the world, are extremely diverse and significant causes of nosocomial infections. On the other hand, a recently discovered strain of *Klebsiella pneumoniae* known as hvKP has gained international attention as a pathogen. Due to a number of improved virulence characteristics, including increased capsule synthesis and siderophore-mediated iron uptake, these hypervirulent strains are more pathogenic than classical strains. Hypervirulent strains are linked to two major capsular serotypes, K1 (more prevalent) and K2, which greatly enhance their pathogenicity and capacity to induce serious invasive infections [22].

Numerous community-acquired infections can be caused by hvKP. The most typical symptom is a pyogenic liver abscess, which can then spread from the liver to other distant locations such as the lungs, eyes, and central nervous system. The organism's strong invasive potential and virulence are demonstrated by its ability to produce primary extrahepatic infections, such as bacteremia, pneumonia, and soft tissue infections, in addition to liver involvement [23]. 

Treatment

Since *Klebsiella pneumoniae *often develops multidrug resistance, the treatment of infections caused by this bacterium depends on the infection site and the antibiotic susceptibility pattern. Fluoroquinolones, aminoglycosides, and third-generation cephalosporins such as ceftriaxone and cefotaxime are commonly used against strains that remain susceptible. Carbapenems, such as Meropenem, Imipenem, and Ertapenem, are frequently the preferred medications for severe infections such as bacteremia or hospital-acquired pneumonia, especially in illnesses brought on by germs that produce ESBL [24].

Alternative or combination therapy is necessary for infections brought on by CRKP. Ceftazidime-avibactam, meropenem-vaborbactam, and imipenem-relebactam are examples of more recent β-lactam/β-lactamase inhibitor combos that have demonstrated action against some germs that produce carbapenemase. Tigecycline, colistin, and amikacin are other medications used to treat MDR infections. These drugs are commonly used in combination therapy to enhance treatment results. To lessen treatment failure and stop the development of new resistance, source handling (such as draining abscesses or removing contaminated catheters), effective infection control strategies, and antimicrobial stewardship are crucial in addition to antibiotic therapy. In general, managing *Klebsiella pneumoniae* infections necessitates cautious antibiotic selection based on susceptibility and culture results, particularly in the age of rising multidrug resistance [22].

Future diagnosis

In order to guarantee early detection and suitable treatment, *Klebsiella pneumoniae *infections will increasingly rely on quick and sophisticated diagnostic technology. Conventional culture techniques are still crucial, but they take a lot of time and can cause delay in clinical judgement. Certain resistance genes, like those that produce carbapenemase, can be quickly identified using contemporary molecular techniques like polymerase chain reaction (PCR) and real-time PCR [25]. Next-generation sequencing and whole-genome sequencing are examples of advanced technologies that offer comprehensive details on virulence factors and antibiotic resistance mechanisms. Furthermore, fast identification of bacterial species from clinical samples is made possible by technologies like MALDI-TOF (matrix-assisted laser desorption/ionization time-of-flight) mass spectrometry. It is anticipated that new methods, such as biosensor-based detection systems and CRISPR-based diagnostics, would increase the speed, sensitivity, and precision of diagnosis. The early detection of MDR germs will be greatly aided by these developments, which will also enhance patient outcomes and infection prevention [26]. 

## Conclusions

Carbapenemase-producing *Klebsiella pneumoniae *is one of the most critical threats to worldwide public health because of its rapid spread, high virulence, and extensive antimicrobial resistance. The organism’s ability to produce a wide range of carbapenemase enzymes, such as KPC, NDM, VIM, IMP, and OXA-48, significantly limits treatment options and is associated with a rise in morbidity, mortality, and healthcare spending. These enzymes, often encoded on mobile genetic elements, facilitate the rapid dissemination of resistance within and across bacterial populations. As the prevalence of multidrug-resistant and hypervirulent strains continues to rise, early and accurate detection becomes essential for proper management of patients and infection prevention. Phenotypic methods, including the modified Hodge test and modified carbapenem inactivation method, remain practical tools in routine clinical microbiology laboratories for detecting carbapenemase production in *Klebsiella pneumoniae* isolates. These methods provide valuable insights into resistance mechanisms and guide appropriate therapeutic decisions. However, with the increasing complexity of resistance patterns, incorporating advanced molecular diagnostics will be crucial for improving detection accuracy and response time. Strengthening antimicrobial stewardship, infection prevention strategies, and global surveillance efforts is vital to controlling the spread of carbapenemase-producing *Klebsiella pneumoniae* and reducing its impact on healthcare systems.
